# Exploring the association between Cerebral small‐vessel diseases and motor symptoms in Parkinson's disease

**DOI:** 10.1002/brb3.1219

**Published:** 2019-02-27

**Authors:** Ying Wan, Wenjian Hu, Jing Gan, Lu Song, Na Wu, Yuzhen Chen, Zhenguo Liu

**Affiliations:** ^1^ Department of Neurology Xinhua Hospital Affiliated to Shanghai JiaoTong University School of Medicine Shanghai China

**Keywords:** association, cerebral small‐vessel diseases, Parkinson's disease, postural instability and gait disability motor phenotype, risk factors

## Abstract

**Objectives:**

to explore the association between cerebral small‐vessel diseases (CSVDs) and motor symptoms in Parkinson's disease (PD).

**Methods:**

137 PD patients were recruited into the study. Detailed motor symptoms, including tremor, rigidity, bradykinesia, and axial impairment, were evaluated using Unified Parkinson's disease Rating Scale (UPDRS). Non‐motor symptoms, including cognition, anxiety, and depression, were evaluated using Montreal Cognitive Assessment (MoCA), Hamilton anxiety scale (HAMA), and Hamilton depression scale (HAMD). Brain MRI was used to assess the subtypes of CSVDs, including lacunes, enlarged perivascular spaces (EPVS), and white matter hyperintensities (WMH). WMH were furtherly divided into deep WMH (DWMH) and periventricular hyperintensities (PVH). The association between CSVDs and motor symptoms was analyzed. Patients were divided into the postural instability and gait disability (PIGD) group and non‐PIGD group. Demographic, clinical and CSVDs variables were compared between the two groups.

**Results:**

CSVDs subtypes were all detected in the participants with different prevalence rates and severity degrees. We found a close association between EPVS in basal ganglia and the tremor score (*p* = 0.032), and between DWMH in the frontal and occipital lobes and the axial motor score (*p* < 0.05) through the spearman and multivariate liner regression analysis. Compared with the non‐PIGD group, the PIGD group demonstrated more serious cognitive impairment and DWMH in the frontal and occipital lobes (*p* < 0.05). The demographic characteristics and vascular risk factors of the PIGD group were not different from those of the non‐PIGD group. Cognitive impairment and DWMH in the frontal lobe were identified to be independent risk factors of PIGD motor phenotype.

**Conclusions:**

We identified a close association between the CSVDs and motor symptoms in PD and DWMH in the frontal lobe was a risk factor of PIGD motor phenotype, which supports the contribution of vascular pathology in PD.

## Abstract 2

Our study provided a fresh data of the comorbid cerebral small‐vessel diseases (CSVDs) in the Chinese Parkinson's disease (PD) patients and supported a close link between the CSVDs burden and motor symptoms in PD. Our study provided the first data of enlarged perivascular spaces in the PD patients. The data confirmed that deep white matter hyperintensities of the frontal lobe as an independent risk factor of postural instability and gait disability motor phenotype.

## 1 INTRODUCTION

Parkinson's disease (PD) is a common neurodegenerative disorder in the elderly people, featured by the pathology of nigrostriatal dopaminergic degeneration. Recently, increasing evidence suggested a close association between the vascular pathology and motor impairments in PD (Bohnen, Müller, & Zarzhevsky, 2011; Malek et al., 2016). Among the motor symptoms, axial motor impairments characterized by the postural and gait dysfunctions were found to be more closely related to the brain white matter hyperintensities (WMH) which is one subtype of Cerebral small vessel diseases (CSVDs) (Ciliz et al., 2018; Lee et al., 2009). The mechanisms underlying these findings are still unclear. Normal posture and gait depend on the elaborate interaction between cortices and the subcortical nuclei (Nutt, Horak, & Bloem, 2011). Thus, WMH induced abnormal interactions between cortices, or between cortices and subcortical nuclei might result in the dysfunction of the Central Neural System (CNS) and abnormal postural and gait in PD. WMH could be anatomically divided into deep WMH (DWMH) and periventricular hyperintensities (PVH). Since the corticocortical and cortical‐subcortical interactions of different brain regions might play different roles in the motor control, we hypothesized that WMH in different brain regions may be associated with various motor impairments in PD patients.

Cerebral small vessel‐diseases (CSVDs) have been reported to be associated with mild parkinsonian signs in aged people (Hatate et al., 2016). In addition to WMH, CSVDs include lacunes and enlarged perivascular spaces (EPVS). The comorbid CSVDs’ impact on the motor symptoms in PD has not been fully clarified. Lacunes have been reported to be frequently observed in PD patients, with a prevalence of nearly 50% (Zhang et al., 2016), however, the association between lacunes and different motor symptoms in PD has not been explored. Moreover, there was no data on the prevalence of EPVS in PD patients or the links between EPVS and motor symptoms in PD.

According to the motor features, PD patients are usually divided into three motor phenotypes: tremor dominant (TD), postural instability, and gait disorder dominant (PIGD), intermediate (ID). PIGD motor phenotype featured by axial motor impairments has been found to be poorly responsive to levodopa treatment. Vascular factors might be involved in the pathophysiology of PIGD motor phenotype (Malek et al., 2016). Clinical data from Chinese PD patients are required to support the viewpoint.

Accordingly, we designed this cross‐sectional study with the purposes to estimate the three CSVDs subtypes in Chinese PD patients and explore its association with motor symptoms in PD. Moreover, we divided the participants into the group of PIGD motor phenotype and non‐PIGD motor phenotype. We hoped to provide a detailed description of the clinical and imaging features of PIGD group and clarify associated risk factors through the logistic regression models.

## 2 PATIENTS AND METHODS

### 2.1 Patients

From January 2013 to January 2017, we recruited 137 PD patients from the Movement disorder clinics of the Department of Neurology of Xinhua Hospital affiliated to Shanghai Jiaotong University School of Medicine. The study was approved by the Research Ethics Committees of Xinhua Hospital affiliated to Shanghai Jiaotong University School of Medicine. All the participants signed written informed consent form. PD patients would be included into this study if they were consistent with all of the following conditions: (a) have been clinically diagnosed with PD; (b) have received dopaminergic treatment for more than half a year before recruited; (c) have been showing clear beneficial response to the dopaminergic treatment. The participants would be excluded from the study if they had at least one of the following conditions: (a) evidence of atypical, secondary or hereditary parkinsonian syndromes; (b) history of the head injury or head trauma; (c) history of a large vessel stroke; (d) contraindications for MRI scanning; (e) inability to receive the study assessment. All participants would receive 3.0T magnetic resonance imaging (MRI) scanning of brain. Those, whose MRI imaging indicated an evidence of brain tumor, hydrocephalus or an atypical parkinsonian disorder, would also be excluded.

### 2.2 Clinical assessment

Demographic characteristics and clinical histories would be acquired through a semi‐structured interview. The clinical histories included the onset age of PD, related medical information as well as the common vascular risk factors of the history of hypertension (defined by the consistent usage of antihypertensive agent or a systolic pressure above 140 mmHg or a diastolic pressure above 90 mmHg demonstrated by repeated examinations before recruited), diabetes mellitus (defined by the history of treatment or a recent record of fasting blood glucose above 126 mg/dl), hyperlipidemia (defined by the history of treatment or total cholesterol level above 200 mg/dl or a low‐density lipoprotein level above 130 mg/dl at the time of presentation), cardiac diseases (defined as a known history of any heart disease, including angina, myocardial infarction, arrhythmia or heart failure), stroke or transient ischemic attack (TIA), and cigarette smoking.

The severity of motor symptoms would be assessed using the Unified Parkinson's Disease Rating Scale (UPDRS). The UPDRS motor part would be furtherly divided into four categories: tremor (UPDRS III, item 20 and 21), rigidity (UPDRS III, item 22), bradykinesia (UPDRS III, items 23–26, and item31), axial motor symptoms (UPDRS III, items 27–30) (Bohnen et al., 2011). The severity of non‐motor symptoms, for example, cognition impairment would be assessed by using the Montreal Cognitive Assessment (MoCA), and the severity of anxiety and depression would be evaluated by the assessment tools of Hamilton anxiety scale (HAMA) and Hamilton depression scale (HAMD). All assessments were conducted by two well‐trained movement disorder neurologists who were blind to the MRI evaluation. In order to acquire a detailed description of clinical and imaging features and associated risk factors of PIGD motor phenotype, all patients would be divided into the PIGD group and non‐PIGD group according to the ratio of the average tremor score and average PIGD score (Jankovic et al., 1990).

### 2.3 MRI evaluation

MRI was conducted using a 3.0 T scanner (GE Signa). The imaging protocol involved T1‐weighted imaging (repetition time [TR] 2200 ms, echo time [TE] 24 ms, section thickness 5 mm) and T2‐weighted fluid‐attenuated inversion recovery (T2‐FLAIR, TR 8500 ms, TE 120 ms, section thickness 5 mm). Structural MRI was analyzed by an experienced observer who was blind to the clinical data. Lacunes were defined as round or ovoid cavities with the diameter from 3 mm to 15 mm, and located in supratentorial structures including the thalamus and basal ganglia. It was characterized by a hypointense lesion and hyperintense rim on FLAIR‐images, according to the corresponding hyper‐and hypo‐intensity on T2‐andT1‐images, respectively. The presence of lacunes would be examined in the basal ganglia and thalamus (Hatate et al., 2016). Enlarged perivascular spaces (EPVS), defined as <3 mm round or linear CSF–isointense lesions along the course of penetrating arteries, were rated on MRI in the basal ganglia and centrum semiovale (Doubal, MacLullich, Ferguson, Dennis, & Wardlaw, 2010). EPVS would be counted in the slice with the highest number. The number of EPVS in one side of the brain would be graded by the following scale applied to standard axial images: 0 if no EPVS, 1 if EPVS ≤ 10, 2 if EPVS = 11–20, 3 if EPVS = 21–40, and 4 if EPVS ≥ 40. The higher score would be used if there was asymmetry between the two sides. The total EPVS score would be acquired by summing up the scores of basal ganglia and centrum semiovale, with the maximum score of 8. WMH, defined as hyperintense lesions on FLAIR‐images, would be assessed with the Scheltens scale (Scheltensa et al., 1993). WMH was divided into two parts: periventricular hyperintensities (PVH) and deep white matter hyperintensities (DWMH). PVH would be assessed in three regions (frontal caps, occipital caps and lateral bands) and graded on a scale of 0–2. DWMH would be assessed in four regions (the frontal, parietal, occipital, and temporal lobe) and graded on a scale of 0–6. The total PVH and DWMH scores would be separately counted by summing up of the score of each region, with the max score of 6 and 24.

### 2.4 Statistics

Descriptive statistics were used as needed. Continuous variables were expressed as mean values and standard deviations, and categorical variables were noted as numbers and percentages. Mann–Whitney *U* test was used to compare the continuous variables in abnormal distribution. χ^2^ test was used to compare the categorical variables. Spearman rank correlation and multivariable liner regression model were used to analyze the association between each motor category and CSVDs variables. Univariate and multivariate logistic regression models were used to identify the independent risk factors of PIGD motor phenotype. Statistical significance was set at *p *<* *0.05, with a two‐tailed approach. Statistical computations were performed by SPSS.

## 3 RESULTS

### 3.1 Characteristics of the study population

A total of 137 PD patients were included in the present study. The demographic and clinical characteristics of the study population were presented in Table 1. The mean age was 68.1 (8.6) years old and 61.3% were males. The average PD duration was 5.2 (4.9) years. Among the vascular risk factors, hypertension was the most common one, with the prevalence of 35.0% whereas the history of stroke or TIA was the least common, with the prevalence of 2.9%. The CSVDs characteristics of the population were shown in Table 2. The prevalence of lacunes in the basal ganglia and thalamus were 43.8% and 20.4%, respectively. The mean total EPVS score was 0.4 (0.5). The mean total PVH score was 3.5 (1.7) and the mean total DWMH score was 4.8 (2.9). The EPVS and WMH scores in different brain regions were presented in Table 2, respectively.

**Table 1:** Clinical characteristics of the study population

	PD patients (*N* = 137)	Group of PIGD type (*N* = 89)	Group of non‐PIGD type (*N* = 38)	*p*
Male *n* (%)^a^^,^ c	84 (61.3%)	60 (61.2%)	24 (61.5%)	0.97
Age, years^b^^,^ d	68.1 (8.6)	68.2 (8.4)	67.8 (9.4)	0.88
Age at onset, y^b^^,^ d	63.0 (9.6)	62.8 (9.8)	63.3 (9.3)	0.71
Hypertension, *n* (%)^a^^,^ c	48 (35.0%)	32 (32.7%)	16 (41.0%)	0.43
Hyperlipidemia, *n* (%)^a^^,^ c	10 (7.3%)	5 (5.1%)	5 (12.8%)	0.23
Diabetes mellitus, *n* (%)^a^^,^ c	15 (10.9%)	12 (12.2%)	3 (7.7%)	0.56
History of stroke or TIA, *n* (%)^a^^,^ c	4 (2.9%)	2 (2.0%)	2 (5.1%)	0.38
History of cardiac disease, *n* (%)^a^^,^ c	9 (6.6%)	6 (6.1%)	3 (7.7%)	0.74
Cigarette smoking, *n* (%)^a^^,^ c	18 (13.1%)	15 (15.3%)	3 (7.7%)	0.23
Duration, years^b^^,^ d	5.2 (4.9)	5.5 (5.3)	4.5 (3.4)	0.66
UPDRS‐II^b^^,^ d	12.31 (7.3)	13.6 (7.8)	9.0 (4.2)	0.002^e^
UPDRS III score^b^^,^ d	24.9 (14.2)	26.5 (15.5)	21.0 (9.5)	0.09
Tremor score^b^^,^ d	3.1 (3.1)	4.8 (2.7)	2.4 (3.8)	<0.001^e^
Rigidity score^b^^,^ d	4.9 (3.8)	5.4 (4.1)	3.8 (2.8)	0.055
Bradykinesia score^b^^,^ d	10.4 (6.8)	11.4 (7.2)	8.0 (4.8)	0.014^e^
Axial motor score^b^^,^ d	4.5 (3.1)	5.2 (3.3)	2.8 (1.4)	<0.001^e^
MoCA score^b^^,^ d	21.0 (4.8)	20.3 (4.9)	23.2 (4.2)	0.003^e^
HAMA score^b^^,^ d	11.0 (9.1)	11.8 (9.5)	9.4 (8.2)	0.28
HAMD score^b^^,^ d	14.9 (12.5)	16.2 (13.9)	12.4 (8.9)	0.43
LED, mg/day^b^^,^ d	560.0 (314)	538.2 (327.4)	606.2 (282.5)	0.31

PIGD: postural instability and gait disability; UPDRS: Unified Parkinson Disease Rating Scale; LED: levodopa equivalent dosage; MoCA: montreal cognitive assessment; HAMA: Hamilton anxiety scale; HAMD: Hamilton depression scale.

a Resulted are presented as number (percentage).

b Resulted are presented as mean (standard error).

c Data were analyzed by χ^2^ test.

d Data were analyzed by Mann‐Whitney *U* test.

e *p* < 0.05 of the comparison between two groups.

**Table 2:** CSVDs characteristics of the study population

	PD patients (*N* = 137)	Group of PIGD type (*N* = 89)	Group of non‐PIGD type (*N* = 38)	*p*
Lacunes in basal ganglia, *n* (%)^a^^,^ c	60 (43.8%)	45 (45.9%)	15 (38.5%)	0.90
Lacunes in thalamus, *n* (%)^a^^,^ c	28 (20.4%)	22 (22.4%)	6 (15.4%)	0.92
Total EPVS score^b^^,^ d	0.4 (0.5)	0.5 (0.7)	0.3 (0.6)	0.10
EPVS score (basal ganglia)^a^^,^ c	0.26 (0.45)	0.3 (0.5)	0.2 (0.4)	0.80
EPVS score (centrum semiovale)^a^^,^ c	0.22 (0.43)	0.3 (0.5)	0.1 (0.3)	0.048^e^
Total PVH score^b^^,^ d	3.5 (1.7)	3.7 (1.7)	3.0 (1.7)	0.84
PVH (frontal caps) score^b^^,^ d	1.2 (0.7)	1.3 (0.6)	1.0 (0.7)	0.30
PVH (occipital caps) score^b^^,^ d	1.0 (0.8)	1.1 (0.8)	0.9 (0.8)	0.68
PVH (lateral bands) score^b^^,^ d	1.3 (0.6)	1.3 (0.6)	1.1 (0.6)	0.72
Total DWMH score^b^^,^ d	4.8 (2.9)	5.1 (3.0)	4.0 (2.7)	0.12
DWMH (frontal lobe) score^b^^,^ d	1.5 (1.1)	1.6 (1.1)	1.1 (1.0)	0.02^e^
DWMH (parietal lobe) score^b^^,^ d	1.6 (1.4)	1.6 (1.4)	1.6 (1.4)	0.98
DWMH (occipital lobe) score^b^^,^ d	0.9 (1.0)	1.0 (1.0)	0.7 (0.9)	0.02^e^
DWMH (temporal lobe) score^b^^,^ d	0.8 (1.0)	0.9 (1.0)	0.5 (0.8)	0.80

PIGD: postural instability and gait disability; CSVDs: cerebral small vessel diseases; EPVS: enlarged perivascular spaces; PVH: periventricular hyperintensities; DWMH: deep white matter hyperintensities.

a Resulted are presented as number (percentage).

b Resulted are presented as mean (standard error).

c Data were analyzed by χ^2^ test.

d Data were analyzed by Mann‐Whitney *U* test.

e *p* < 0.05 of the comparison between two groups.

### 3.2 CSVDs and Parkinson's motor symptoms

The association between each CSVDs subtype and the four motor categories was analyzed by spearman correlation analysis. The results showed that the EPVS score in the region of basal ganglia closely correlated to the tremor score (*p* = 0.03). The DWMH scores in the regions of the frontal lobe (*p* = 0.03) and occipital lobe (*p* = 0.02) closely correlated to the axial motor score. Meanwhile, among all the clinical variables, the tremor and axial motor scores were both linked to the PD duration. In the multivariable linear analysis adjusting for PD duration, a significant association was still found between the tremor score and the EPVS score (basal ganglia), and between the axial motor score and the DWMH scores (the frontal and occipital lobes) (Table S1). However, the bradykinesia and rigidity scores correlated to none of the CSVDs variables (Table 3). There was no statistical difference in the scores of the four motor categories between PD patients with lacunes and without lacunes in basal ganglia or thalamus.

**Table 3:** Association between each CSVD subtype and each motor category of UPDRS‐III

	Tremor score	Rigidity score	Bradykinesia score	Axial motor score
EPVS score (basal ganglia)	0.18 (0.03)^a^	0.03 (0.72)	0.11 (0.19)	0.12 (0.17)
EPVS score (centrum semiovale)	0.05 (0.58)	0.08 (0.36)	0.14 (0.09)	0.12 (0.16)
PVH (frontal caps) score	0.01 (0.94)	−0.11 (0.18)	0.03 (0.76)	0.12 (0.15)
PVH (occipital caps) score	0.03 (0.69)	0.001 (0.99)	0.06 (0.52)	0.06 (0.48)
PVH (lateral bands) score	−0.11 (0.20)	−0.10 (0.23)	0.000 (0.99)	0.10 (0.23)
DWMH (frontal lobe) score	0.003 (0.97)	−0.07 (0.39)	0.04 (0.62)	0.18 (0.03)^a^
DWMH (parietal lobe) score	0.08 (0.36)	0.014 (0.87)	0.08 (0.35)	0.09 (0.27)
DWMH (occipital lobe) score	0.001 (0.99)	0.14 (0.09)	0.33 (0.70)	0.20 (0.02)^a^
DWMH (temporal lobe) score	−0.07 (0.42)	−0.08 (0.36)	0.04 (0.67)	0.08 (0.37)

*Note.*Results are presented as *r* (*p* value).

EPVS: enlarged perivascular spaces; PVH: periventricular hyperintensities; DWMH: deep white matter hyperintensities.

a *p* < 0.05 of the correlation between variables.

### 3.3 CSVDs and PIGD motor phenotype

Compared with the group of non‐PIGD motor phenotype, the group of PIGD motor phenotype had higher scores in the categories of tremor, bradykinesia and axial motor impairments, a higher UPDRS‐II score and a lower MoCA score (*p* < 0.05) (Table 1). Moreover, the group of PIGD motor phenotype had a higher EPVS score in the region of centrum semiovale and higher DWMH scores in the regions of the frontal lobe and occipital lobe than the group of non‐PIGD motor phenotype (*p* < 0.05) (Table 2). No difference was found in the demographic characteristics or other clinical and CSVDs variables between the two groups.

Variables of the MoCA score, EPVS score and the DWMH score with significant difference between groups were furtherly included into the univariate logistic regression analysis. The results showed that PIGD motor phenotype closely correlated to a lower MoCA score (OR = 0.87, 95% CI = 0.79–0.95, *p* = 0.003) and a higher DWMH score in the frontal lobe (OR = 1.59, 95% CI = 1.08–2.34, *p* = 0.019) (Table 4). In the multivariable logistic regression analysis, a lower MoCA score (OR = 0.87, 95% CI = 0.79–0.95, *p* = 0.003) and a higher DWMH score in the frontal lobe (OR = 1.62, 95% CI = 1.07–2.43, *p* = 0.021) were finally identified to be independent risk factors of PIGD motor phenotype.

**Table 4:** Univariate logistic regression for determining risk factors of the PIGD motor phenotype

	OR	95% CI	*p*
MoCA score	0.87	0.79–0.95	0.003^a^
EPVS score (centrum semiovale)	2.97	0.98–9.03	0.055
DWMH score (frontal lobe)	1.59	1.08–2.34	0.019^a^
DWMH score (occipital lobe)	1.34	0.88–2.03	0.17

PIGD: postural instability and gait disability; MoCA: montreal cognitive assessment; EPVS: enlarged perivascular spaces; DWMH: deep white matter hyperintensities; OR: odds ratio; CI: confidence interval.

a *p* < 0.05.

## 4 DISCUSSION

Our study provided fresh data of the comorbid CSVDs in the Chinese PD patients. The data support a close link between the CSVDs burden and motor symptoms in PD.

We identified a close association between axial motor impairments and DWMH of the frontal and occipital lobes. We also found that PIGD motor phenotype featured by axial motor impairments carried more serious DWMH in the two regions than the non‐PIGD motor phenotype did. It indicates that the lesions of nerve fiber tracts in the two regions may play a crucial role in axial motor impairments.

Axial motor functions, especially the posture and gait, are a series of extraordinarily complex motor behaviors consisting of primary elements: locomotion, balance and the ability to adapt to the surrounding environment. All these complex behaviors closely depend on the elaborate coordination of the whole CNS, in which cortex (especially the frontal cortex), basal ganglia, thalamus, and cerebellum are considered as the most essential regions. The normal function of these CNS regions and their related intact interaction is indispensable for normal posture, gait, and balance (Nutt et al., 2011). We propose that DWMH of the frontal lobe may interrupt the corticocortical and cortico‐basal ganglia/thalamus interactions, and lead to the aberrant signal transmission as well as the occurrence of axial motor impairments in PD. Recently, a longitudinal study also found a positive relationship between the frontal WMH and the severity of PIGD symptoms, and the comorbid frontal WMH increased the likelihood for non‐PIGD PD patients at baseline to develop further PIGD symptoms during 5 years of follow‐ups (Lenfeldt, Holmlund, Larsson, Birgander, & Forsgren, 2016). Occipital cortex, the visual processing center of the brain, has been demonstrated to be associated with freezing of gait in PD (Guan et al., 2017). It is noteworthy that the interactions between the occipital cortex and the other cortices are crucial in maintaining coordinated posture and gait. Additionally, the occipitoparietal pathway, which consists of reciprocal projections to the prefrontal and premotor cortices, participates in mapping the spatial relationships of objects and provides supports for the visually guided movements (Kravitz, Saleem, Baker, & Mishkin, 2011). PD patients were reported to be more dependent on the external stimulus (especially visual cues) for movement initiation and coordinated walking (Lewis, Byblow, & Walt, 2000). In our opinion, that DWMH of the occipital lobe may interrupt the occipital cortical interaction with the frontal cortex and reduce the visual information to the frontal cortex, leading to the impaired ability to adapt the body to the surrounding environment in the static or dynamic status. We found that the severity of axial motor impairments was not associated with the PVH score. It indicates that there may be some anatomical and functional distinctions between DWMH and PVH (Griffanti et al., 2018; Kim, MacFall, & Payne, 2008). However, no related reports have been found in PD yet.

Our findings showed that lacunes were more commonly detected in the basal ganglia than thalamus, with a high prevalence of 43.8%. The prevalence rate was slightly lower than the previous study (Zhang et al., 2016). Asymptomatic lacunar infarction (LI) or lacunes are most commonly detected in the basal ganglia. Previous studies found that LI in basal ganglia or other regions was not associated with the severity of motor symptoms (Song, Kim, Cho, & Chung, 2013; Zhang et al., 2016). In our study, lacunes in the two targeted regions correlated to none of the four categories of motor symptoms or the PIGD motor phenotype. However, in Zhang's 1‐year follow‐up study (Zhang et al., 2017), PD patients with striatal LI presented a more rapid motor function decline than PD patients without striatal LI. Accordingly, the comorbid striatal LI or lacunes might be an index of a rapid motor function decline, for which more evidence will be needed.

Our study provided the first data of EPVS in the PD patients. We found that over one‐third of PD patients had EPVS at a slight degree. Moreover, the EPVS score in the region of basal ganglia significantly correlated to the severity of tremor and the EPVS score in the region of centrum semiovale was much higher in PD patients of PIGD motor phenotype than that in patients of the non PIGD motor phenotype. Currently, clinical studies have mainly focused on investigating the association between EPVS and cognitive impairment, poor blood pressure control or disease worsening in patients with dementia (Wang et al., 2018), stroke (Liu et al., 2018), or systematic inflammation (Cavallari et al., 2018). The relevant studies proposed that a higher EPVS severity degree might result in impaired cerebrovascular reactivity, blood‐brain barrier dysfunction and perivascular inflammation in the diseases (Brown et al., 2018). No attention has been paid on the association between EPVS and PD. We speculate that similar pathophysiological changes caused by EPVS may contribute to the association between EPVS and the specific motor symptoms in PD. More data would be needed to support our viewpoint.

We found that patients of PIGD motor phenotype demonstrated a more serious cognitive impairment and heavier DWMH burdens, compared to the patients of non‐PIGD motor phenotype. Our findings support that there might be some heterogenicity in different motor phenotypes of PD. Recent studies showed that, compared with patients of TD motor phenotype, patients of PIGD motor phenotype presented more comprehensive, serious and faster disease progression in the multiple fields of cognition, autonomic function and sleep (van der Heeden et al., 2016; Huang et al., 2018), most of which were dopamine resistant symptoms. In our study, DWMH was identified to be a risk factor of PIGD motor phenotype. It strongly supports that the vascular pathology might be closely involved in the pathophysiology of PIGD motor phenotype. Accordingly, the non‐dopaminergic intervention of vascular remodeling in the brain might be a novel treatment in PIGD motor phenotype in the future.

There are some limitations in this study. Cerebral microbleeds (CMBs), another subtype of CSVDs, were reported to be closely linked to cognitive impairment rather than motor symptoms in PD (Ham et al., 2014). Therefore, CMSs were not examined in our study. In addition, this was a cross‐sectional study and the findings would be confirmed by a longitudinal evaluation which is in progress.

## CONFLICT OF INTEREST

The authors declared that the study was conducted in the absence of any commercial or financial relationships that could be construed as a potential conflict of interest.

## AUTHOR CONTRIBUTION

Y W., ZG L. designed the study. WJ H., J G. finished clinical evaluations of the PD patients. L S. finished imaging evaluation of the study. YZ C. and N W. finished the data input and analyzed the data. Y W. and WJ H. wrote the paper. ZG L revised the paper.
