# Supplementary material for: Exploring the association between Cerebral small‐vessel diseases and motor symptoms in Parkinson's disease
Source: Brain Behav. 2019 Feb 27;9(4):e01219. doi: 10.1002/brb3.1219 (PMC6456802; doi:10.1002/brb3.1219)
Supplement: Supplementary file 1 [file BRB3-9-e01219-s001.docx]

Supplementary table

Table 1 Multivariable linear regression for the motor symptoms’ association with CSVD variables

|  | Tremor score | | | | Axial motor score | | | |
| --- | --- | --- | --- | --- | --- | --- | --- | --- |
|  | β^a^ | SE^b^ | t | p | β^a^ | SE^b^ | t | p |
| CSVD variables | | | | | | | | |
| EPVS (basal ganglia) score | 0.23 | 0.53 | 2.96 | 0.004* | / |  |  |  |
| DWMH score (frontal lobe) | / |  |  |  | 0.24 | 0.23 | 3.00 | 0.003* |
| DWMH score (occipital lobe) | / |  |  |  | 0.18 | 0.25 | 2.26 | 0.025* |

CSVDs= Cerebral small vessel diseases; EPVS=Enlarged perivascular space; DWMH= deep white matter hyperintensities ^a^:Standardized coefficients; ^b^: Standard Error; *: p<0.05. The multivariable linear regression was done in the condition that motor symptoms of PD duration were controlled for.
